# *TAp73* modulates proliferation and ferroptosis in mammary epithelial cells

**DOI:** 10.3389/fcell.2025.1532910

**Published:** 2025-04-03

**Authors:** Wenqiang Sun, Hanjun Ren, Le Chen, Bingfei Zhang, Liping Mei, Jiaqi Wen, Yilu Zhang, Jiaqi Li, Yongping Yan, Songjia Lai

**Affiliations:** ^1^ Farm Animal Genetic Resources Exploration and Innovation Key Laboratory of Sichuan Province, Sichuan Agricultural University, Ya’an, China; ^2^ State Key Laboratory of Swine and Poultry Breeding Industry, College of Animal Science and Technology, Sichuan Agricultural University, Ya’an, China; ^3^ Key Laboratory of Livestock and Poultry Multi-Omics, Ministry of Agriculture and Rural Affairs, College of Animal Science and Technology, Sichuan Agricultural University, Ya’an, China

**Keywords:** *TAp73*, mammary epithelial cells, cell death, ferroptosis, proliferation

## Abstract

**Introduction:**

*TAp73*, a transcriptionally active isoform of the *p73* gene, is essential for epithelial tissue development. Ferroptosis, a regulated form of cell death characterized by lipid peroxidation and reactive oxygen species (ROS) accumulation, has been increasingly studied in recent years. However, its role in epithelial cells and the regulatory function of *TAp73* in this context remain poorly understood.

**Methods:**

We investigated the role of *TAp73* in epithelial cell proliferation and ferroptosis using ectopic overexpression and RNA interference approaches. Cell proliferation was assessed through colony formation and DNA synthesis assays. Ferroptosis was induced using RSL3, and the effects were evaluated by measuring cell viability, ROS levels, and the expression of ferroptosis-associated genes *PTGS2* and TFRC.

**Results:**

*TAp73* overexpression significantly increased *p21* expression, suppressed colony formation and DNA synthesis, thereby inhibiting cell proliferation. In contrast, *TAp73* knockdown reduced *p21* levels and enhanced cell proliferation. RSL3 treatment induced a dose-dependent increase in cell death and ROS accumulation, confirming the susceptibility of epithelial cells to ferroptosis. Furthermore, *TAp73* overexpression enhanced RSL3-induced ferroptosis by upregulating *PTGS2* and *TFRC*, while *TAp73* knockdown diminished their expression, reducing oxidative stress and lipid peroxidation.

**Conclusion:**

*TAp73* acts as a dual regulator of epithelial cell fate by inhibiting proliferation and promoting ferroptosis. These findings reveal a novel role for *TAp73* in epithelial cell biology and suggest potential therapeutic targets for diseases involving epithelial cell death.

## Introduction

The mammary ducts, comprising two main cell populations—basal and luminal cells—the mammary gland undergoes dynamic structural remodeling throughout the lactation cycle. In late pregnancy, luminal progenitors differentiate terminally to form secretory alveoli, which are specialized for milk biosynthesis and secretion (Macias and Hinck, 2012). Thus, understanding the dynamic development of mammary epithelial cells is crucial.

Previous studies have underscored the significance of specific genetic factors in mammary gland development. Zhang et al. established that conditional deletion of *TAp73* – the transcriptionally active isoform encoded by the *TP73* gene – in murine mammary epithelia disrupts alveolar morphogenesis. Notably, genetic ablation of the oncogenic Δ*Np73* isoform rescues morphological defects arising from PUMA or p21 deficiency, revealing an antagonistic interplay between *p73* isoforms during alveologenesis (Zhang et al., 2015). Yan et al. further reported that *TAp73* knockdown results in irregular, non-cavitary alveoli and induces epithelial-mesenchymal transition by modulating the expression of E-cadherin, β-catenin, and laminin (Zhang et al., 2012). These findings highlight the necessity of *p73* for normal mammary gland development.

Ferroptosis, an iron-dependent regulated cell death pathway driven by lipid peroxidation, has emerged as a key determinant of tissue homeostasis and disease pathogenesis (Dixon et al., 2012; Li et al., 2020). This process can be triggered by various factors and involves multiple signaling pathways. For instance, the inhibition of GPX4 activity reduces cellular antioxidant capacity, leading to increased intracellular ROS and the onset of ferroptosis (Ursini and Maiorino, 2020). However, it is still rarely reported whether mammary epithelial cells can undergo ferroptosis.

Central to this regulatory network, p53 acts as a key ferroptosis sensitizer by transcriptionally repressing SLC7A11, the cystine/glutamate antiporter, leading to glutathione depletion and facilitating lethal lipid peroxidation (Jiang et al., 2015). Structurally homologous to *p53*, *p73* participates in overlapping molecular networks governing proliferation and death, yet exhibits distinct context-dependent functionalities (Lokshin et al., 2007; Jost et al., 1997). The diverse isoforms of *p73*, resulting from selective promoter usage and alternative splicing, include *TAp73*, which exerts tumor-suppressive effects, and *ΔNp73*, which has oncogenic properties (Irwin et al., 2003). Research indicates that *TAp73* can induce ferroptosis in lung cancer cells by suppressing CDO1 expression, leading to increased ROS accumulation (Zhang et al., 2023). This functional conservation, coupled with mammary-specific *p73* expression patterns, strongly implicates p73 isoforms as putative modulators of ferroptotic signaling in lactating epithelia.

Although previous studies have focused on human mammary epithelial cells, our study employs bovine mammary epithelial cells (BMECs) as a model to investigate p73-mediated ferroptosis and proliferation. BMECs serve as a valuable model due to their physiological relevance in lactation biology and mammary gland development in livestock, which has direct implications for dairy production and animal health. Additionally, bovine models provide unique insights into mammary gland function in species with extended lactation cycles, making them particularly relevant for studying long-term epithelial cell dynamics.

Our study aims to investigate, for the first time, whether *TAp73* can regulate both the proliferation and ferroptosis of BMECs. By exploring the expression changes of ferroptosis-related genes and assessing cell proliferation and death under conditions of *TAp73* ectopic expression and knockdown, we seek to elucidate the role of *TAp73* in these fundamental cellular processes. This research could provide novel insights into the molecular mechanisms governing mammary gland development and function.

## Materials and methods

### Cell culture

The Bovine mammary epithelial cell line (BMECs, MAC-T), provided by Dr. Chunlei Zhang of Jiangsu Normal University (Xuzhou, China), was cultured in DMEM/F12 medium (Gibco, CA, United States) supplemented with 10% fetal bovine serum (FBS, Gibco, CA, United States). Cells were maintained at 37°C in a 5% CO_2_ incubator. The cells were grown in T-75 cell culture flasks until they reached 70%–80% confluence and were sub-cultured using a 0.25% trypsin/EDTA solution (Gibco, CA, United States). For all experiments, cells from passages 5–6 were used to ensure consistency and optimal cell viability. Cell viability was assessed routinely using the Trypan Blue exclusion method, with viability consistently maintained above 95%. The culture duration for each experiment was approximately 24–48 h, depending on the experimental design. Additional culture conditions, such as media changes every 24–48 h, were strictly followed to ensure cell health and experimental reproducibility.

### Plasmid construction

To overexpress TAp73, we engineered a mammalian expression vector, pcDNA3.1-TAp73, by subcloning the full-length bovine TAp73 cDNA, obtained from TSINGKE (China), into the pcDNA3.1 vector (Invitrogen, Shanghai, China).

### Cell transfection

The TAp73 ectopic expression plasmid (pcDNA3.1-TAp73) or TAp73-targeting siRNA (final concentration of 20 nM) was transfected into cells using Lipofectamine 3000 (Invitrogen, Carlsbad, CA), following the manufacturer’s instructions. Empty pcDNA3.1 vector and scrambled siRNA were used as respective controls. For ectopic expression experiments, cells were transfected with 2 μg of plasmid DNA per well and harvested 24 h post-transfection. In siRNA-mediated knockdown experiments, cells were maintained for 72 h, with the medium replaced every 24 h. To assess transfection efficiency, TAp73 mRNA levels were quantified by qRT-PCR, demonstrating over a 100-fold induction in the ectopic expression group and approximately 50% knockdown in siRNA-treated cells compared to controls. Furthermore, successful transfection was validated by analyzing p21 mRNA expression via qRT-PCR, as p21 is a well-established transcriptional target of TAp73.

### Real-time quantitative PCR analysis

Total RNA was isolated from BEMCs using TRIzol reagent (Aidlab, Beijing, China) and quantified with a NanoDrop 2000 spectrophotometer (Thermo Scientific, Wilmington, DE, United States). After treating with DNase I to eliminate genomic DNA, cDNA synthesis was conducted using the PrimeScript RT Master Mix kit (Vazyme, Nanjing, China). RT-qPCR was then carried out on a ForeQuant F4 Sequence detection system with SYBR®Premix Ex Taq™ (Foregene, Chengdu, China). The PCR cycling protocol included an initial step at 95°C for 10 min, followed by 40 cycles at 95°C for 10 s and 60°C for 40 s, during which fluorescence signals were collected. All primer sequences can be found in Table 1. The expression levels of the genes were normalized to glyceraldehyde-3-phosphate dehydrogenase (GAPDH) and beta-actin (β-actin) and calculated using the 2^−ΔΔCT^ method (Livak and Schmittgen, 2001).

**TABLE 1:** Primer sequences.

Primer name	Sequence
GAPDH	GCGCCAAGAGGGTCATCATCTCTG CATAAGTCCCTCCACGATGCCAAAG
β-actin	GCCCATCTATGAGGGGTACGC CTCCTTGATGTCACGGACGATTTC
TFRC	TTGGATTTATGATTGGCTACTTGGG AATATGCGAGGTACTCCAGGGAGTTG
PTGS2	TCTTCCTCCTGTGCCTGATGACTGC ACATCAGATTTGTGCCCTGGGGATC
p73	ACCTCATCCGTGTGGAGGGCAAC CACCTGTCCGTCCCGTGTCTCC

### 5-Ethynyl-2′-deoxyuridine (EdU) assay

Cell proliferation was assessed using the BeyoClick™ EdU Cell Proliferation Kit with Alexa Fluor 555 (Beyotime, China). Cells were seeded in a 12-well plate and incubated with EdU working solution (1:1000 dilution) for 1 h at 37°C in a humidified atmosphere with 5% CO_2_. After incubation, cells were fixed with methanol for 15 min and permeabilized with 0.5% Triton X-100 for 5 min. They were then incubated with Click reaction solution for 30 min and stained with DAPI for 10 min. Images were captured from at least 12 random fields per group using an Olympus IX73 microscope (Olympus, Tokyo, Japan). Cell counting was conducted using ImageJ software.

### Colony formation assay

Control and pcDNA3.1-TAp7α or si-TAp73 cells were seeded in six-well plates (approximately 1000 cells per well, in triplicate) and cultured for 2 weeks. Colonies were fixed with a methanol/glacial acetic acid solution (7:1) and stained with crystal violet. Colony density was quantified using ImageJ software with the ColonyArea plugin, as described previously (Guzman et al., 2014).

### Measurement of ROS levels

Reactive oxygen species (ROS) levels were measured using a commercially available ROS detection kit (Beyotime, China), following the manufacturer’s instructions. Treated cells were incubated with the fluorescent probe DCFH-DA (1:1000 dilution) at 37°C for 30 min. Cells were then fixed with 4% paraformaldehyde for 10 min, washed three times with PBS, and stained with DAPI for 10 min. The stained cells were visualized under an Olympus IX73 microscope (Olympus, Tokyo, Japan).

#### CCK-8 assay

BMECs were seeded in 96-well plates and treated with RSL3. After the treatment, 10 µL of CCK-8 solution (Solarbio Life Sciences, Beijing, China) was added to each well, and the plates were incubated for 2 h. The optical density (OD) at 450 nm was measured using a Varioskan LUX spectrophotometer (Thermo Scientific, Waltham, MA, United States). The half-maximal inhibitory concentration (IC50) of RSL3 was then determined based on the CCK-8 assay results.

#### LDH content

BMECs were treated with RSL3 for 24 h, while the control group received DMSO treatment. After incubation, the supernatant was collected by centrifugation at 1,000 g for 5 min, and lactate dehydrogenase (LDH) levels were measured using an LDH cytotoxicity assay kit (Solarbio Life Sciences, Beijing, China).

#### Statistical analysis

Data are presented as mean ± standard deviation (SD). For each experiment, the sample size is explicitly stated in the figure legends. Each experiment was repeated independently at least three times to ensure reproducibility. Normality of data distribution was assessed using the Shapiro-Wilk test. For comparisons between two groups, the student’s t-test was applied when data met assumptions of normality and homogeneity of variance; For comparisons among more than two groups, one-way analysis of variance (ANOVA) with Tukey’s *post hoc* test was performed for normally distributed data. Statistical significance was defined as p < 0.05. All analyses were conducted using SPSS 17.0 statistical software.

## Results

### Ectopic expression of TAp73 can inhibit BMECs proliferation

**FIGURE 1:**
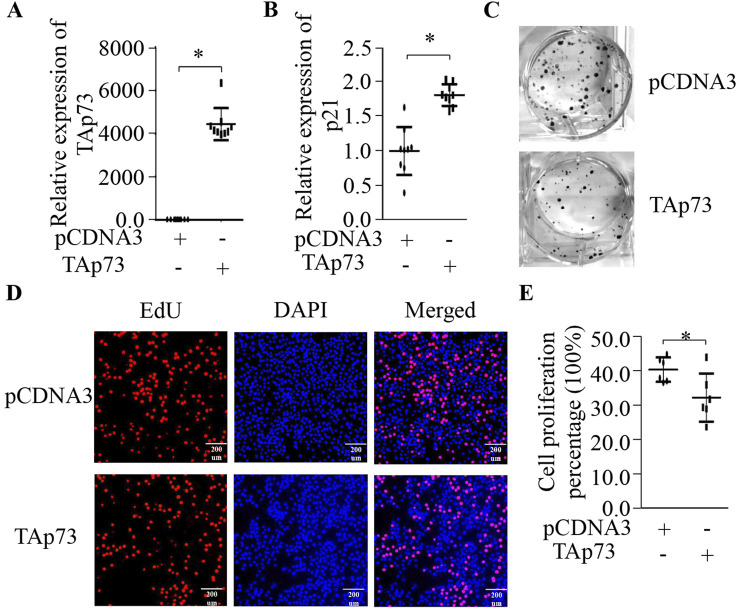
Effect of ectopic expression of TAp73 on BMECs proliferation. **(A)** Relative expression levels of TAp73 in BMECs following TAp73 plasmid transfection. **(B)** Relative expression levels of p21 in BMECs following TAp73 plasmid transfection **(C)** A colony formation assay was performed following TAp73 plasmid transfection. **(D, E)** EdU assay was performed following TAp73 plasmid transfection. Three biological replicates and three technical replicates were performed for the qPCR analysis. The colony formation and EdU assays were conducted with six replicates. Data are presented as mean ± SD. *p < 0.05.

To investigate whether TAp73 can regulate BMECs, we ectopic expressionTAp73 and quantified its canonical transcriptional target p21 (Figure 1A). Our findings revealed that TAp73 significantly upregulates p21 expression (Figure 1B) (p < 0.05). Following this, we conducted colony formation and EdU assays to examine the effects on cell proliferation. The results showed that the colony formation assay demonstrated a substantial decrease in colony numbers in TAp73-overexpressing cells compared to control cells, indicating inhibited cell growth (Figure 1C). Similarly, the EdU assay, which measures DNA synthesis as an indicator of cell proliferation, showed a significant reduction in the number of proliferating cells upon TAp73 ectopic expression (Figures 1D,E). These findings collectively support the role of TAp73 in modulating cell cycle progression and proliferation in BMECs, highlighting its potential as a key regulatory factor in these cells.

### Knock-down TAp73 can accelerates BMECs proliferation

**FIGURE 2:**
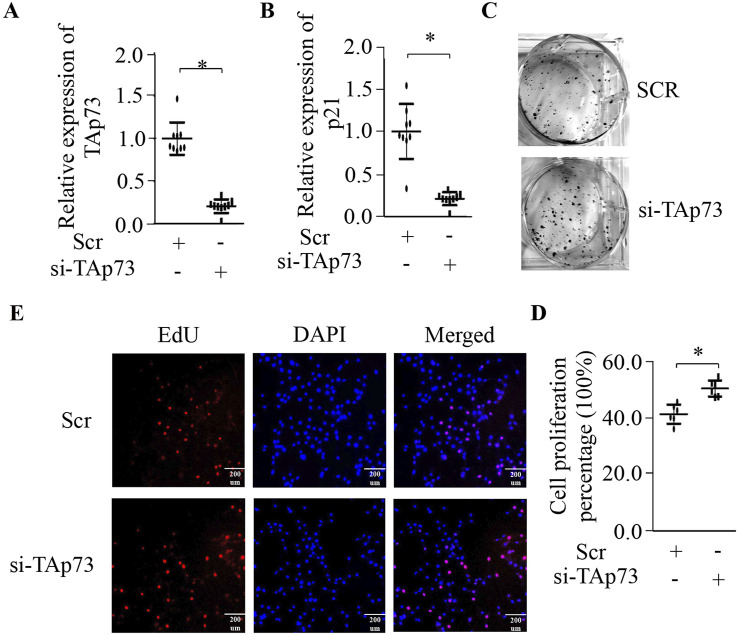
Effect of knockdown of TAp73 on BMECs proliferation. **(A)** Relative expression levels of TAp73 in BMECs following TAp73 siRNA transfection. **(B)** Relative expression levels of p21 in BMECs following TAp73 siRNA transfection. **(C)** A colony formation assay was performed following TAp73 siRNA transfection. **(D, E)** EdU assay was performed following TAp73 siRNA transfection. Three biological replicates and three technical replicates were performed for the qPCR analysis. The colony formation and EdU assays were conducted with six replicates. Data are presented as mean ± SD. *p < 0.05.

To further validate the influence of TAp73 on BMECs proliferation, we performed transfection with TAp73 siRNA to suppress the expression of endogenous TAp73. RNAi-mediated TAp73 silencing (confirmed in Figure 2A, p < 0.05) reduced p21 expression (p < 0.05; Figure 2B), attenuating cell cycle constraints. We then conducted colony formation and EdU assays to assess the effects on cell proliferation. The colony formation assay showed a substantial increase in colony numbers in TAp73 knockdown cells compared to control cells, indicating promoted cell growth (Figure 2C). Similarly, the EdU assay demonstrated a significant increase in the number of proliferating cells upon TAp73 knockdown (Figures 2D,E). These findings further emphasize the role of TAp73 in modulating cell cycle progression and proliferation in BMECs, underscoring its potential as a key regulatory factor in these cells.

### BMECs can induce ferroptosis

**FIGURE 3:**
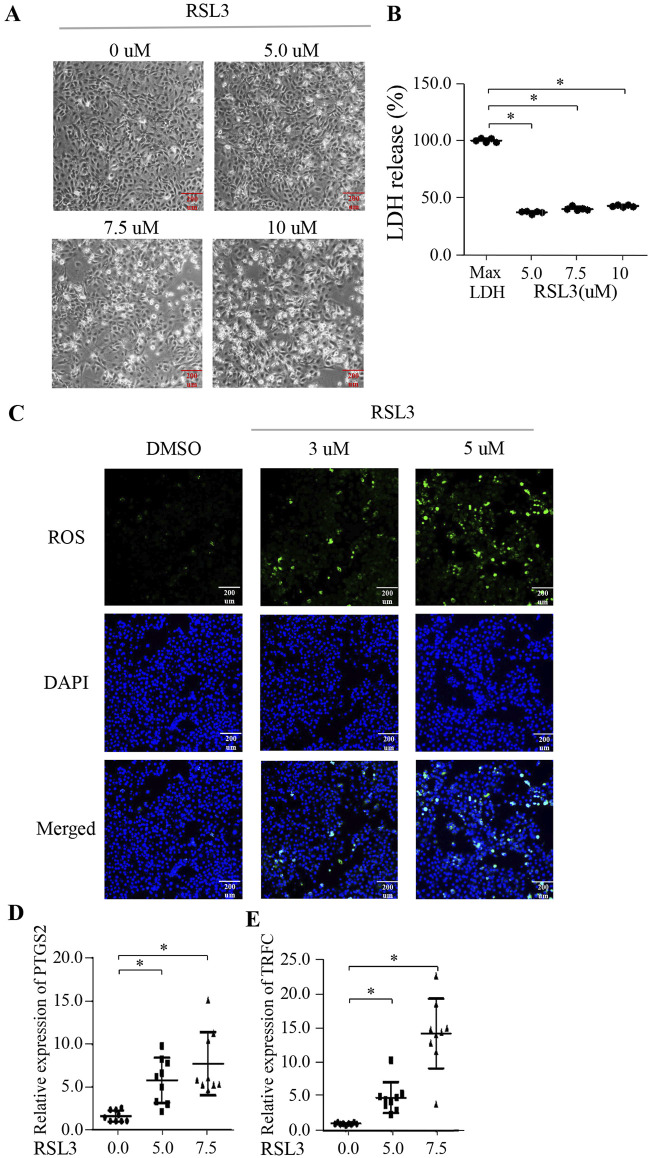
BMECs can undergo ferroptosis. **(A)** BMECs were treated with various concentrations of RSL3 (0, 5, 7.5, 10 μM) for 24 h, and representative microscopic images were taken to show cell morphology. **(B)** LDH content of BMECs cells treated with RSL3 (0, 5, 7.5, 10 μM) for 24 h. **(C)** Intracellular ROS levels were measured using the fluorescent probe DCFH-DA in BMECs cells treated with RSL3 (0, 3, 5 μM) for 24 h. **(D, E)** Relative expression levels of PTGS2 and TRFC in BMECs cells treated with RSL3 (0, 5, 7.5 μM) for 24 h. Three biological replicates and three technical replicates were performed for the qPCR analysis. The LDH, colony formation, and EdU assays were conducted with six biological replicates each. Data are presented as mean ± SD. *p < 0.05.

To explore the potential for ferroptosis in BMECs, we treated these cells with the classical ferroptosis inducer, RSL3. Our results demonstrated a dose-dependent increase in cell death following RSL3 treatment (Figure 3A). Notably, RSL3 treatment led to a significant, dose-dependent release of LDH, further confirming cell death (Figure 3B). The IC50 value for RSL3 in BMECs was calculated to be 6.315 µM (Supplementary Figure S1A). Additionally, there was a corresponding dose-dependent accumulation of reactive oxygen species (ROS) within the cells (Figure 3C), indicating that BMECs can undergo ferroptosis when induced by RSL3. Additionally, we assessed the expression of ferroptosis-related genes PTGS2 and TRFC, observing a significant elevation in their expression levels post-RSL3 treatment (Figures 3D,E) (p < 0.05). These findings collectively suggest that BMECs are capable of undergoing ferroptosis.

### TAp73 is involved in the regulation of ferroptosis in BMECs

**FIGURE 4:**
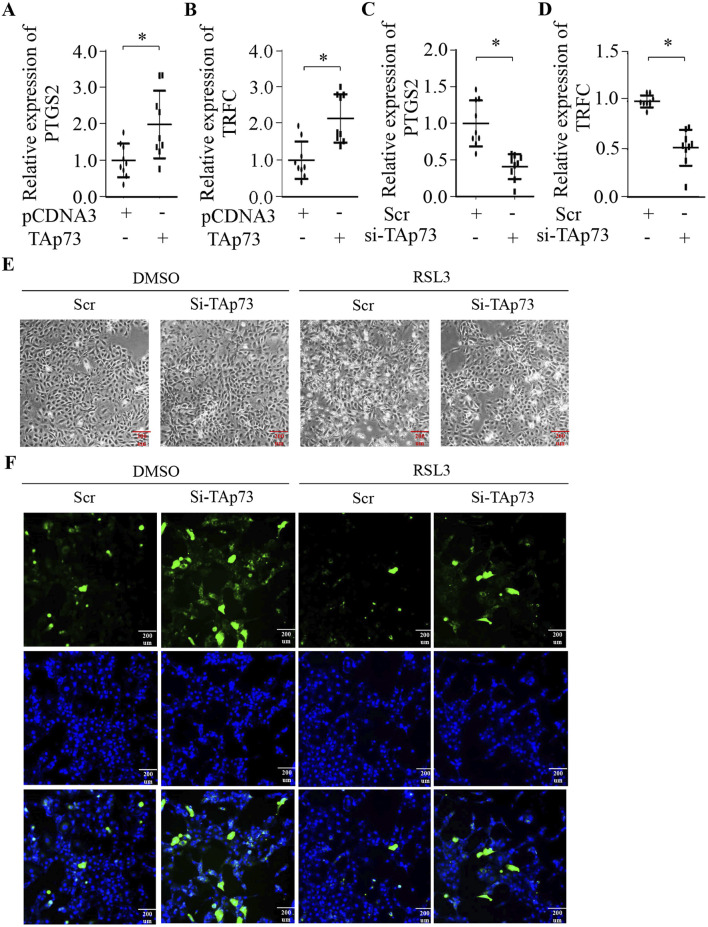
TAp73 is Involved in the Regulation of Ferroptosis in BEMCs. **(A, B)** Relative expression levels of PTGS2, and TRFC in BEMCs following TAp73 plasmid transfection. **(C, D)** Relative expression levels of PTGS2 and TRFC in BEMCs cells following si-TAp73 transfection. **(E)** BEMCs cells were treated with RSL3, si-TAp73, or their combination, and representative microscopic images were taken to show cell morphology. **(F)** Intracellular ROS levels were measured using the fluorescent probe DCFH-DA in BEMCs cells treated with RSL3, si-TAp73, or their combination. Three biological replicates and three technical replicates were employed in the qPCR analysis to ensure data reliability and reproducibility. Data are presented as mean ± SD. *p < 0.05.

To explore whether TAp73 can regulate ferroptosis in BMECs, we first examined the expression of ferroptosis-related genes. Our findings revealed that ectopic expression of TAp73 significantly promotes the expression of PTGS2 and TRFC, which are key genes involved in the ferroptosis pathway (Figures 4A,B). This suggests that TAp73 might enhance the susceptibility of BMECs to ferroptosis. Conversely, knockdown of TAp73 resulted in a marked decrease in the expression levels of PTGS2 and TRFC (Figures 4C,D) (p < 0.05), indicating a potential protective effect against ferroptosis. To further validate the role of TAp73 in ferroptosis, we treated BMECs with the ferroptosis inducer RSL3 while simultaneously knocking down TAp73 expression. The results demonstrated that TAp73 knockdown significantly attenuated RSL3-induced ferroptosis, as evidenced by reduced cell death and a decrease in ROS accumulation (Figures 3E,F). This indicates that TAp73 knockdown can mitigate the oxidative stress and cellular damage typically associated with ferroptosis. Additionally, the reduction in ROS levels upon TAp73 knockdown was accompanied by decreased lipid peroxidation. Further supporting the role of TAp73 in regulating ferroptosis in BMECs.

## Discussion

Our investigation into the role of p73, a member of the p53 family, in regulating BMECs revealed significant insights. The TP73 gene encodes two functionally antagonistic isoforms, *TAp73* and *ΔNp73*, which are produced through alternative promoter usage and C-terminal splicing (Rufini et al., 2011). Functionally divergent, these isoforms govern opposing cellular outcomes: *TAp73*, a transcriptionally active p53 homolog mediating cell cycle arrest, whereas ΔNp73 acts as a dominant-negative regulator over TAp73, possessing its own distinct transcriptional activities (Jost et al., 1997; Irwin et al., 2003). Known for its dual role in tumor suppression and promotion, p73 is also critical in the development and differentiation of specific tissues and organs, such as neuronal differentiation, the development of the central nervous and olfactory systems, and is highly expressed in airway ciliated columnar cells and the myoepithelial and basal cells of the salivary gland (Rufini et al., 2011; Yang et al., 2000; Lee et al., 2007).

Previous studies have highlighted *TAp*73’s necessity for proper mammary epithelial acinar formation and its ability to suppress cell migration and invasion by regulating the expression of E-cadherin and other epithelial-mesenchymal transition (EMT) markers (Zhang et al., 2012). To interrogate its functional role in BMECs, we employed gain- and loss-of-function approaches targeting *TAp*73. Our findings showed that ectopic expression of *TAp73* significantly upregulates *p21* expression (Figure 1B) (p < 0.05). This upregulation was associated with a substantial decrease in colony formation and a significant reduction in DNA synthesis, as demonstrated by the EdU assay (Figures 1C–E). These results suggest that *TAp73* inhibits cell proliferation in BMECs, highlighting its potential as a key regulatory factor. Conversely, RNAi-mediated *TAp73* silencing suppressed *p21* expression (Figure 2B) (p < 0.05). The colony formation assay showed a substantial increase in colony numbers, and the EdU assay demonstrated a significant increase in the number of proliferating cells upon *TAp73* knockdown, indicating promoted cell growth (Figures 2C–E). These findings further emphasize *TAp*73’s role in modulating cell cycle progression and proliferation in BMECs, underscoring its importance as a regulatory factor.

Ferroptosis, an iron-catalyzed form of regulated cell death driven by lethal lipid peroxidation (Ursini and Maiorino, 2020; Yang and Stockwell, 2016), has emerged as a pivotal mechanism implicated in various physiological and pathological processes, including cancer, neurodegeneration, and ischemia-reperfusion injury (Lei et al., 2022; Vitalakumar et al., 2021; Zhao et al., 2021). At the core of ferroptosis execution is the inactivation of glutathione peroxidase 4 (GPX4), which disrupts redox homeostasis by allowing lipid peroxide accumulation through Fenton chemistry (Shimada et al., 2016), ultimately culminating in membrane rupture and lytic cell death (Seibt et al., 2019). Although extensively studied in other cell types, the occurrence of ferroptosis in bovine mammary epithelial cells rarely explored.

Our study aimed to explore the potential for ferroptosis in BMECs by treating these cells with the classical ferroptosis inducer, RSL3 (Shintoku et al., 2017). The results demonstrated a dose-dependent increase in cell death following RSL3 treatment, indicating that BMECs are susceptible to ferroptosis induction. This was further supported by the corresponding dose-dependent accumulation of ROS within the cells, a hallmark of ferroptosis. Consistent with ferroptosis biomarkers, RSL3 treatment significantly upregulated PTGS2, a sensor of lipid peroxides, and TFRC, a key regulator of iron uptake (Chen et al., 2021; Feng et al., 2020), further confirming the occurrence of ferroptosis in BMECs. These findings collectively suggest that BMECs are capable of undergoing ferroptosis when exposed to appropriate inducers like RSL3. The ability of BMECs to undergo ferroptosis expands our understanding of the cellular responses in mammary epithelial cells and highlights the potential implications for dairy cow health.

Having established BMECs’ ferroptotic susceptibility, we next interrogated whether TAp73 modulates this death pathway. Given its dual role in oxidative stress responses and cell fate determination, we hypothesized that TAp73 acts as a ferroptosis rheostat in mammary epithelia. TAp73 gain-of-function robustly upregulated PTGS2and TFRC (p < 0.05; Figures 4A,B), priming cells for lipid peroxidation. Conversely, TAp73 loss-of-function suppressed both markers (Figures 4C,D), attenuating ferroptotic priming. The increased expression of PTGS2 and TRFC in *TAp*73-overexpressing cells indicates a heightened state of oxidative stress and lipid peroxidation, which are hallmarks of ferroptosis. Conversely, knocking down *TAp73* resulted in a marked reduction in the expression levels of these ferroptosis-related genes, highlighting a potential protective role against ferroptosis. This downregulation of PTGS2 and TRFC suggests that the absence of *TAp73* may help maintain cellular homeostasis by mitigating oxidative stress and preventing the accumulation of toxic lipid peroxides. The observed decrease in these gene expressions upon *TAp73* knockdown underscores the importance of *TAp73* in the ferroptosis process. To further substantiate the involvement of *TAp73* in ferroptosis, we conducted experiments where BMECs were treated with the ferroptosis inducer RSL3 while simultaneously knocking down *TAp73* expression. The results were compelling: *TAp73* knockdown significantly alleviated RSL3-induced ferroptosis, as evidenced by a notable reduction in cell death and a decrease in ROS accumulation. This indicates that *TAp73* knockdown can effectively mitigate the oxidative stress and cellular damage typically associated with ferroptosis, thereby conferring a protective effect against this form of cell death. Moreover, the reduction in ROS levels observed upon *TAp73* knockdown was accompanied by decreased lipid peroxidation, further supporting the role of *TAp73* in regulating ferroptosis. The attenuation of lipid peroxidation is particularly significant, as it underscores the mechanism by which *TAp73* influences ferroptosis: by modulating the cellular oxidative state and lipid metabolism. These findings collectively highlight *TAp73* as a key regulatory factor in ferroptosis, modulating the susceptibility of BMECs to oxidative stress and lipid peroxidation.

## Conclusion

This study underscores the critical role of *TAp73* in regulating both proliferation and ferroptosis in BMECs. Ectopic expression of *TAp73* significantly reducing cell proliferation, while *TAp73* knockdown increased cell growth. We confirmed that BMECs can undergo ferroptosis when treated with RSL3, with dose-dependent increases in cell death and ROS accumulation. Notably, *TAp73* modulates this process: its ectopic expression enhanced PTGS2 and TRFC expression, increasing susceptibility to ferroptosis, while its knockdown mitigated these effects, reducing oxidative stress and lipid peroxidation. These findings highlight *TAp*73’s dual regulatory role in BMECs, impacting both cell proliferation and ferroptosis. Understanding these mechanisms offers new therapeutic strategies for managing oxidative stress and enhancing dairy cow health and productivity.

## Data Availability

The original contributions presented in the study are included in the article/Supplementary Material, further inquiries can be directed to the corresponding author.
