# Supplementary figures and images for: TAp73 modulates proliferation and ferroptosis in mammary epithelial cells
Source: Front Cell Dev Biol. 2025 Apr 3;13:1532910. doi: 10.3389/fcell.2025.1532910 (PMC12003338; doi:10.3389/fcell.2025.1532910)

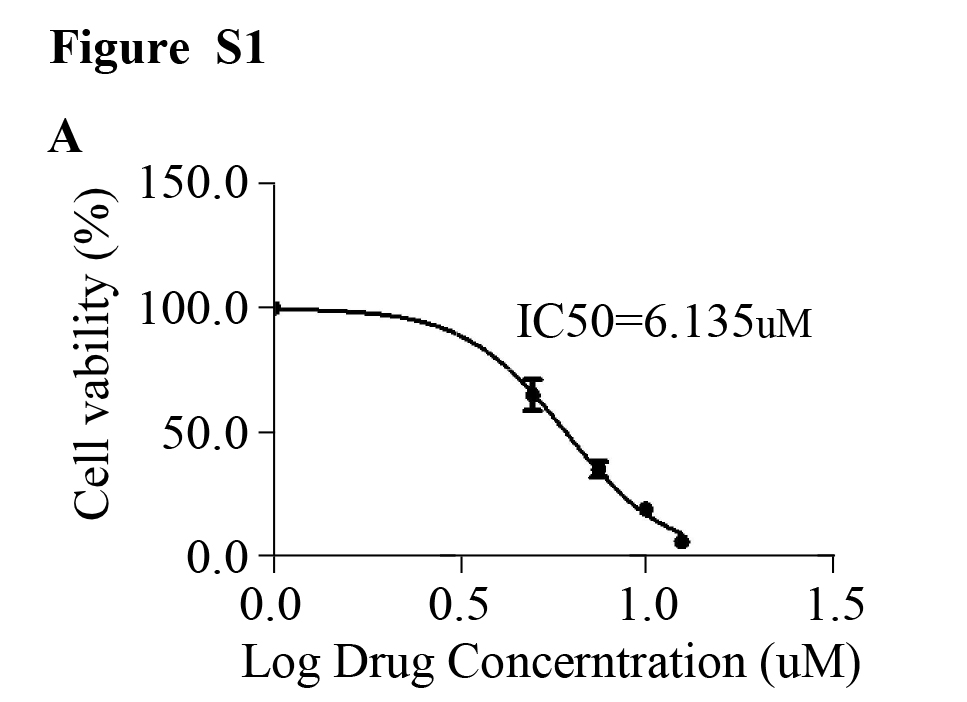

Supplement: Supplementary file 1 [file Image1.jpeg]
